# Subliminal emotional pictures are capable of modulating early cerebral responses to pain in fibromyalgia

**DOI:** 10.1371/journal.pone.0217909

**Published:** 2019-06-05

**Authors:** Irene Peláez, David Ferrera, Paloma Barjola, Roberto Fernandes, Francisco Mercado

**Affiliations:** Unit of Clinical Psychology, Faculty of Health Sciences, Rey Juan Carlos University, Madrid, Spain; RWTH Aachen, GERMANY

## Abstract

Pain experience involves a complex relationship between sensory and both emotional and cognitive factors, which appear to be mediated by different neural pathways. Previous evidence has shown that whereas conscious processing of unpleasant stimuli enhances pain perception, the influence of emotions on pain under unaware conditions is much less known. The need to better characterise the relationship between pain processing and emotional factors is crucial for dealing with chronic pain conditions. Therefore, the present study aimed to explore the neural correlates relating to the influence of visual masking emotional stimulation on the processing of painful stimuli in chronic pain patients suffering from fibromyalgia (FM). Twenty FM and 22 healthy control (HC) women participated in the study. The experimental masking paradigm consisted of a rapid succession of two types of stimuli, where a masked picture (neutral, negative or pain-related) was followed by a laser stimulus (painful or not painful). LEP activity was recorded at sixty scalp electrodes. An LEP-amplitude approach was used to quantify the main cerebral waves linked to pain response. ANOVAs indicated that the posterior regions of the P1 component were sensitive to experimental manipulation (p<0.05). Specifically, FM patients showed higher amplitudes to painful stimuli preceded by pain-related pictures compared with painful trials preceded by other emotional pictures. The FM group also showed greater amplitudes than those in the HC group in P2a and P2b waves. In addition to the scalp data, at the neural level the posterior cingulate cortex, lingual gyrus and insular cortex showed higher activation in the FM group than in the HC group. Our findings show an early cerebral modulation of pain (as reflected by the P1) in FM patients, suggesting that only pain-related information, even when it is unconsciously perceived, is capable to enhance exogenous (automatic) attention, increasing the neural activity involved in processing painful stimulation. Further research is needed to fully understand unconscious emotional influences on pain in fibromyalgia.

## Introduction

Pain is generally understood as a subjective and unique experience, usually accompanied by an unpleasant emotional experience [1]. In healthy states, the main function of the pain processing system is to either prevent or deal with physical harm. However, during chronic pain conditions, as in the case of fibromyalgia, pain loses its protective meaning [2]. Although, the origin of this syndrome is still unknown and under debate, growing scientific evidence has suggested that its symptomatology is mediated by central pain-processing mechanisms [3–5], leading to the appearance of widespread and diffuse musculoskeletal pain as the main symptom [3,6]. Such dysfunctional regulation of pain could be modulated by affective and cognitive factors, in particular those relating to negative emotions or pain-related information [7,8].

It is well documented that emotion can modulate pain perception [9–12]. Specifically, whereas negative emotions have been associated with both an increase in the pain experienced and a decrease in pain tolerance, positive ones lead to the opposite pattern [13,14]. Rhudy and coworkers [11] presented their subjects with a series of pictures varying in emotional valence (i.e., unpleasant, neutral and pleasant) while electrical stimulation was delivered to the left ankle. They found that only the unpleasant pictures had the ability to enhance pain perception. Similarly, pain tolerance decreases [10,15] through the use of different kinds of negative emotional or pain-related contexts (e.g., words or faces). The experimental evidence in patients with chronic pain, generally indicates that negative emotions or pain-related information exacerbate the experience of pain, but the opposite effects with positive emotions are unclear [8,16–19].

The activation of a memory network for pain has been proposed as a potential explanation for this modulation [20,21]. In the case of chronic pain patients, repeated exposure to pain could establish and consolidate a pain memory network [16]. Such experimental evidence has been reported even when individuals were unaware of the stimulus [22]. Several investigations using a subliminal priming paradigm have indicated decreased pain tolerance when participants are exposed to words associated with health problems (e.g. wound, infection). These results may be an important element in explaining somatic complaints with no observable body pathology in chronic pain syndromes. Given that the activation of pain memories could occur under unconscious processes [20,23], this mechanism for inputting information in the cognitive system might influence different behaviours such as symptom experiencing (i.e. pain) without conscious awareness [21]. However, research on the emotional modulation of pain using experimental subliminal paradigms remains scarce, and its results mixed and contradictory. Indeed, whereas a couple of studies found no emotional modulation of pain [24,25], another group reported higher rates of pain perception when somatic stimulation was unconsciously primed by a pain-related word compared with a neutral one [21,22]. Additionally, a recent study conducted by our group confirmed emotional modulation effects on pain perception. The unpleasant content of subliminal pictures, however, generated lower pain perception and higher reaction times to painful stimulation compared with neutral images [26], in contrast to the pattern indicated by previous evidence. Until now, there have been no experiments with FM patients exploring the emotional modulation of pain by subliminal paradigms.

One important issue that could help to clarify these mixed results relates to the kind of emotional stimulation used to prime. The previous literature indicates that pictorial and non-pictorial (words) stimuli have different power to modulate pain processing [25], emotional pictures being both a more arousing and intrusive prime than words [27–29] and one with more ecological value [30]. Additionally, high-temporal resolution brain techniques, such as event-related potentials (ERPs), could be an excellent option for exploring rapid processes such as those involved in emotional priming effects. In particular, ERPs elicited by CO_2_ laser stimulation (laser-evoked potential, or LEP) have been shown to be good indices of pain perception. The study of these processes in patients suffering from chronic pain could help to clarify whether subliminal emotional information may influence pain processing and its perception in such patients. Enhanced amplitudes of LEPs (N2-P2 wave complex) have been reported in FM patients in response to painful stimulation [31,32]; but other aspects, such as emotional context, might also modulate the amplitude of these LEPs. Some studies found that amplitudes of N2 in response to a painful stimulus were higher when it was preceded by negative rather than positive pictures [14,33]. Others, however, failed to find emotional modulation on LEPs in chronic pain patients [34]. Therefore, the present research aimed to explore neural correlates related to the influence of visual masking emotional stimulation on the processing of a painful stimulus (CO_2_ laser stimulation) in patients with FM, by means of LEP methodology. We expected that unpleasant and pain-related pictures would produce an increase in LEP amplitudes (P2/N2 components), even under a condition of unawareness processing.

## Methods

### Participants

A total of fifty-six right-handed women (29 healthy control (HC) subjects and 27 FM patients) took part in the experiment. All participants were aged between 33 and 63 years. Patients fulfilled the 1990 American College of Rheumatology (ACR) diagnostic criteria for FM [35]. Various rheumatologists belonging to the public hospitals of the Community of Madrid carried out the diagnoses of FM. Finally, only data from 42 (22 HC subjects and 20 FM patients) of the 56 who started the experiment were analysed and included in the study, as will be explained later (p.11, EEG recording and pre-processing section). Patients were recruited from the Fibromyalgia and Chronic Fatigue Syndrome Association (AFINSYFACRO) and from the Fibromyalgia Association of Pinto (AFAP). HC participants were recruited by means of both emailed and public advertisements located on campus. The sample of HC participants was made up in such a way as to allow matching for age and education level with patients. No differences were found when the ages (t = 0.65, p = .52) and educational levels (t = -1.05, p = .30) of both groups were compared. Most FM patients were taking analgesics or NSAIDS (nonsteroidal anti-inflammatory drugs). Patients who were taking medication (47.82%; low-dose of benzodiazepines or SSRI) continued to do so because of both medical prescription and ethical considerations. Neurological disease or disorders that impair cognitive functions, psychosis and substance abuse/dependence were set as exclusion criteria, so participants with these medical conditions were excluded from the study. All participants had normal or corrected-to-normal eyesight. The socio-demographic and psychological measures of patients whose data were finally processed are shown in Table 1, along with information about their medication.

**Table 1:** Socio-demographic and psychological measures of fibromyalgia and healthy control groups.

Variables	Mean (SD)		Group effect	
	Healthy control participants	Fibromyalgia patients	Statistic, *t* or *x*^2^	*P*-value
Age (years)	49.90 (8.84)	48.68 (10.29)	0.415	.681
Education				
Elementary studies (%)	17.39	26.08	-0.711	.477
Middle level (%)	30.43	43.47	-0.875	.378
University-level studies (%)	52.17	30.43	1.445	.147
Medication				
Analgesics (%)	0.00	17.39	2.017	.043
NSAIDS (%)	0.00	17.39	2.017	.043
Tricyclics (%)	0.00	0.00	-	-
SSRI (%)	0.00	60.86	4.298	< .001
Benzodiazepines (%)	0.00	17.39	2.017	.043
Others* (%)	30.43	43.47	-0.875	.378
Time elapsed since diagnosis (months)	-	135.13 (73.28)		
VAS pain 1	0.93 (2.09)	4.03 (2.17)	4.695	< .001
VAS pain 2	1.39 (2.09)	5.15 (2.06)	5.857	< .001
VAS fatigue 1	1.57 (2.49)	4.70 (2.02)	4.442	< .001
VAS fatigue 2	2.36 (3.00)	4.58 (2.63)	2.526	.016
Fibromyalgia Impact Questionnaire (total score)	-	61.20 (18.23)		
Spielberger State Anxiety Inventory (STAI-Trait)	37.36 (29.87)	60.30 (28.29)	2.548	.015
Spielberger State Anxiety Inventory (STAI-State)	24.64 (18.92)	39.35 (20.37)	2.427	.020
Pain Catastrophizing Scale (total score)	27.18 (22.34)	43.75 (24.77)	2.279	.028
Beck's Depression Inventory (total score)	5.27 (6.35)	17.30 (5.97)	6.322	< .001
Fear of Pain Questionnaire (total score)	71.91 (21.64)	68.70 (30.27)	-0.398	.693
Tampa Scale for kinesiophobia (total score)	28.73 (11.21)	38.65 (6.04)	3.516	.001

Mean and standard deviations (in parenthesis) of age, education, percentage of participants (HC and FM) taking medication and time elapsed since diagnosis of FM patients. The scores of the self-report instruments were also included.

*Other medication: antihistamines, antibiotics, statins, antihypertensives and replacement hormones.

Participants gave written informed consent for their involvement in the experiment. The Rey Juan Carlos University Research Ethics Board approved the study according to the requirements of this committee. Several self-report instruments were administered to the participants just before starting the experiment. These were two different Visual Analogue Scales (VAS) for assessing both pain perception and fatigue, and the state form of the State-Trait Anxiety Inventory (STAI). At the end of the experiment both VAS were administered again, along with the rest of the self-report questionnaires. The whole sample filled out the STAI [36], the Pain Catastrophizing Scale (PCS) [37], Beck's Depression Inventory (BDI) [38], the Fear of Pain Questionnaire (FPQ-III) [39] and the Tampa Scale for Kinesiophobia [40]. Only FM patients filled out the Fibromyalgia Impact Questionnaire, FIQ [41], a questionnaire to assess their current health and functional status.

### Stimuli and procedure

The Gentask module of the STIM2 package (NeuroScanInc) was used as the software for the stimuli presentation and data acquisition. It includes a dedicated visual system and a four-button response pad for data collection. The experimental paradigm consisted of two types of stimuli. Each trial included a masked emotional picture followed by a laser stimulus. Three types of emotional pictures were presented to participants: neutral (N), arousing-negative (A-) and pain-related (P). All images were matched in size (61, 64° width x 49, 48° height, visual angle degrees) and brightness. Sixty pictures representing A-, P and N emotions were used, and each picture was presented four times. Forty of the pictures belonging to the A- and N emotional categories were selected from the International Affective Picture System (IAPS) [42], according to normative ratings of valence and arousal. Picture numbers from the IAPS database were as follows for the N stimuli: 5510, 7000, 7002, 7004, 7006, 7009, 7025, 7041, 7050, 7059, 7080, 7090, 7100, 7150, 7175, 7224, 7235, 7242, 7491, 7950; and for the A- stimuli: 1052, 1201, 1525, 1930, 2703, 2717, 2811, 3230, 6250, 6313, 6510, 6550, 6570, 6940, 7380, 9250, 9300, 9495, 9571, 9910. For the P category, 20 pictures was selected from the Photograph Series of Daily Activities Scale (PHODA) [43]. PHODA was developed as a diagnostic tool for determining the perceived harmfulness of different physical activities and movements. From this scale the following pictures were selected: 003, 06, 08, 016, 18, 23, 025, 028, 28, 036, 040, 046, 56, 62, 082, 82, hurken, ladderschoen, sprigenvanmuur and stofzuigen.

**Fig 1:**
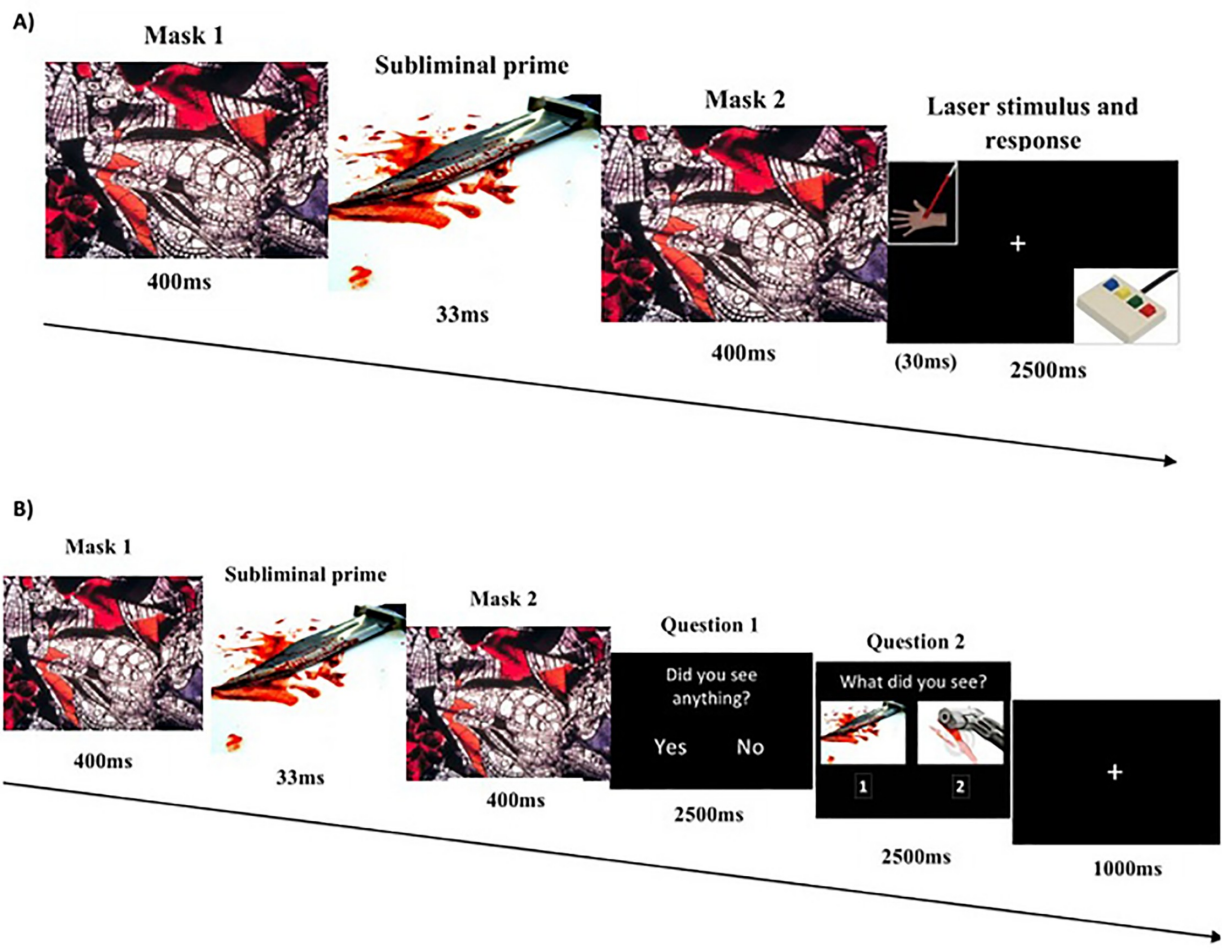
**Schematic representation of the experimental sequences described in the main text for A) the visual masking task, and B) the picture detection test.** A) An example of an A- trial is presented. B) An example of a picture detection test of the same trial (A-)―the sequence of each trial was identical to the one used in the experimental task except for the two questions displayed at the end of each trial: 1) the first, ‘*Did you see anything*?*’* 2) the second, ‘*What did you see*?*’* This question was displayed along with two different pictures: one was the emotional masking picture (belonging to the emotional picture set used in the experimental session); the other was a comparable picture in both emotional category and visual characteristics (shape and colours) to the experimental stimulation (for additional details see [26]).

As described in a previous study [26], in order to ensure that the emotional pictures were not consciously perceived, a forward and backward masking procedure was used (see Fig 1A for full details). The presentation of emotional stimuli followed a semi-random order, such that there were never more than two consecutive trials of the same emotional category.

The experimental session was carried out in a light and sound-attenuated room, in which subjects were seated facing a 19" flat-panel monitor (refresh rate 60Hz) connected to the STIM2 system, at a distance of 60 cm. Participants were instructed to look continuously at the centre of the screen where the visual stimulation was presented. As mentioned above, after the appearance of the emotional masked pictures, laser stimuli were presented in rapid succession (see Fig 1A). Laser stimulation was delivered over the dorsum of participants’ non-dominant hand, the central region being avoided as recommended by previous studies [44]. This stimulation was applied using a CO_2_ laser (Neurolas, Electronic Engineering; wavelength of 10.6 μm) with a power of 9 watts and a duration of 30ms. The laser pulse was set at two intensities: infra-threshold level (non-painful stimulus: it was never perceived as painful by subjects) and supra-threshold level (painful stimulus: it was always perceived as painful). These two intensities were selected for each subject before the experimental session, using the method of limits as in previous studies [16,26]. Infra- and supra-threshold stimuli were delivered via a mean beam diameter of 2.8 mm (density = 30.70 mJ/mm^2^) and 4 mm (density = 21 mJ/mm^2^), respectively. The smaller the diameter, the more painful the stimulus. To avoid nociceptor sensitisation, habituation, skin damage and fatigue of the hand, laser stimulation was shifted about 2 cm after each trial and the stimuli were applied in a semi-random way so that there were never more than two consecutive trials of the same somatosensory category. Finally, to prevent participants seeing the laser beam direction and to avoid distractions, the hand was inserted into a box that was only open at the top. Subjects and experimenters wore protective goggles during all phases of the experimental procedure.

Participants were informed that both laser intensities might be used during the experiment, but never something stronger than they had felt in the previous session. At the end of each trial, they were asked to report the intensity of pain perceived from the laser stimulation, as quickly as possible, by pressing a button on a device with four numbers where ‘1’ corresponded to no pain sensation, ‘2’ to moderate pain, ‘3’ to intense pain and ‘4’ to very intense pain. A total of 240 trials (80 for each emotional category) were performed in which half of the laser stimuli were applied above the pain threshold (painful stimulus) and half below (innocuous stimulus). Combining the three types of emotional pictures and the two intensities of laser stimulation, six experimental conditions of 40 trials each were configured: negative picture followed by painful stimulus (A- Pain), negative picture/innocuous stimulus (A- NoPain), neutral picture/painful stimulus (N Pain), neutral picture/innocuous (N NoPain), pain-related picture/painful stimulus (P Pain) and pain-related picture/innocuous (P NoPain).

The inter-trial interval was set at 3500ms. The task was divided into six blocks of 40 trials each, and after each block participants were offered an optional short break (1–2 mins per break) to minimise fatigue. The entire experimental task lasted 14 minutes. All participants were instructed to perform a practice block in order to familiarise themselves with the experimental task. This block consisted of 20 trials containing 10 N images (different from those used during the task) presented during the appearance of painful and non-painful stimuli.

#### EEG recording and pre-processing

Brain electrical activity was recorded using an electrode cap (ElectroCap International) with 60 homogeneously distributed scalp electrodes. All electrodes were referenced to mastoids. Vertical and horizontal eye movements were monitored through an electrooculographic (EOG) recording. Electrodes were located infra- and supra-orbitally (vertical EOG) as well as at the left and right orbital rim (horizontal EOG). Electrode impedances were kept below 5 kΩ. An online bandpass filter from 0.1 to 40 Hz (3 dB point’s for−6 B/octave roll-off) was applied for the recording amplifiers. Further, data were digitally filtered using a 30 Hz 24 dB/octave low-pass filter. Channels continuously digitised the data at a sampling rate of 250 Hz throughout the entire recording session. Off-line pre-processing was performed using Brain Vision Analyzer software (Brain Products). The continuous recording was divided into 1200ms epochs for each trial, beginning 200ms before stimulus onset. EOG-artifact removal was carried out according to the procedure described by Gratton and coworkers [45]. Baseline correction and EEG visual inspection was also carried out, eliminating epochs with artifacts for further analyses. Data from twelve participants were removed from further analyses because of the high rate of artifact-contaminated trials (over 35%). This artifact rejection procedure led to an average admission of 66.2% (mean = 26.67; SD = 4.53) A- Pain, 68.2% (mean = 27.67; SD = 4.17) A- NoPain, 68.1% (mean = 27.69; SD = 4.16) N Pain, 68.3% (mean = 27.67; SD = 4.31) N NoPain, 67.3% (mean = 27.33; SD = 3.95) P Pain and 72.7% (M = 29.69; SD = 4.38) P NoPain trials. LEP averages were categorised according to each type of stimulus (3 types of emotional pictures x 2 levels of laser stimulus).

#### Picture detection test and emotional picture assessment

After the experimental task, participants were required to perform a forced-choice task [28] to check whether subliminal emotional pictures were indeed shown under the awareness threshold. Before starting this test, participants were informed of the existence of masked images. The forced-choice task was also applied using the Gentask module of the STIM2 package, in accordance with the sequence shown in Fig 1B (for more details see [26]). Therefore, participants were instructed to say in each trial whether they consciously perceived the masked picture and to decide in which location on the screen that masked picture was displayed: on the left or on the right side. The order of presentation for the 120 trials (20 pictures for each of the three emotional categories, repeated twice: one in the left-hand position and the other one in the right-hand position) was pseudo-randomised, so no more than three consecutive trials of the same emotional category or location were shown. Analyses of the extent of stimuli awareness were carried out using an objective threshold for unawareness defined by an identification procedure in which if the stimulus was perceived by the subject in no more than 50% (at chance) of cases [46], according to Signal Detection Theory (SDT) [47] it is unlikely that there was conscious awareness of the stimulus (d´ = 0). Any responses given after 2500ms and omissions were not taken into account in these analyses.

Finally, to confirm whether the emotional pictures had the a priori assumed valence and arousal levels, participants were asked to rate them on a bi-dimensional scaling test (valence: from 1–unpleasant to 5–pleasant; and arousal: from 1–very relaxing to 5–very arousing). Both rating scales were presented at the same time on the screen during the image presentation. Participants made their ratings by selecting their preferred option on the display with the mouse. The assessments given by the participants on these two affective dimensions of visual stimuli are displayed in the results section in Table 2.

**Table 2:** Valence and Arousal relating to the three emotional picture categories.

	N	A-	P
Subjective ratings			
Valence FM	3.28 (0.28)	1.18 (0.19)	2.88 (0.47)
Valence HC	3.37 (0.37)	1.29 (0.34)	3.40 (0.55)
Arousal FM	2.78 (0.30)	4.64 (0.40)	3.27 (0.42)
Arousal HC	2.84 (0.43)	4.51 (0.57)	3.27 (0.52)

Means and standard deviations (in parenthesis) of subjective responses to each of the three types of emotional picture stimuli (neutral–N, negative–A- and pain related–P). Scores for valence and arousal of emotional stimulation varied from 1 (low pleasure, low arousal) to 5 (high pleasure, high arousal).

### Statistical analysis

#### Control and behavioural analyses

To check for possible differences between both laser intensities (infra- and supra-threshold levels) used for each group of participants a t-test for independent samples was conducted.

Participants’ assessments of the values of valence and arousal for the emotional images were analysed using a repeated-measures ANOVA with Emotion (N, A-, P), and Group (FM patients and HC participants) as factors. Post hoc comparisons were made to determine the significance of pairwise contrasts, using the Bonferroni test (alpha < .05).

To test the influence of masked emotional pictures on behavioural performance with respect to group of participants, pain intensity rating (PR) and reaction times (RTs) to laser stimulus were analysed. In the case of RTs, we carried out outlier analyses. Responses above 2500ms or below 200ms were identified in order to be omitted from the analyses. This procedure led to an average admission of 84.4% (M = 34.12, SD = 4.84) A-Pain, 85.3% (M = 34.19, SD = 5.50) A- NoPain, 84.3% (M = 34, SD = 5.57) N Pain, 83.9% (M = 33.81, SD = 5.34) N NoPain, 84.6% (M = 34.17, SD = 6.16) P Pain and 87% (M = 35.02, SD = 5.73) P NoPain trials. Repeated measures ANOVAs exmaining RTs and PR as dependent variables and Emotion (three levels: N, A-, P), Laser stimulus (two levels: Pain and NoPain) and Group (two levels: FM and HC participants) as factors, were carried out. Where necessary, Greenhouse-Geisser (GG) correction was applied to adjust the degrees of freedom of the F ratios and to overcome sphericity violations. Bonferroni adjustment (alpha = .05) was conducted for follow-up contrasts to control for Type I error rate (reported p-values reflect probabilities after Bonferroni correction). A significance level of .05 (two-tailed) was used for all statistical analyses where significant contrast. Effect sizes were computed using the partial eta-square (ƞ^2^_p_) method. Finally, possible relationships between PR and RTs and clinical variables (STAI, BDI and PCS) were examined by means of regression analyses. These variables have been described as important factors related to attention on pain, affecting performance in a concurrent task [4,48,49]. Also, it has been reported that in highly anxious individuals, threat-related information can rapidly capture their attention even without conscious processing of the stimuli [50]. The possible effect of medication on PR and RTs within the FM group was tested for, using a one-way analysis of variance model including patients using and not using particular medications (separately for analgesics, NSAIDS, tricyclics, SSRI and benzodiazepines). All statistical analyses were carried out using IBM SPSS Statistics (version 22).

#### LEP analysis: Detection and quantification

Temporal principal component analysis (tPCA) performed using a covariance matrix was applied to detect and quantify the LEP components explaining most of the brain electrical activity variance. This technique is strongly recommended for these kinds of tasks because it avoids the subjectivity of selecting time windows for component analyses based only on a visual inspection of grand-averaged LEPs; this can lead to several types of misinterpretation, especially when high-density montages are employed (see [51], for a more detailed description of tPCA procedure and advantages). The waveform recorded at a site on the scalp over a period of several hundreds of milliseconds represents a complex superposition of different overlapping electrical potentials. In this sense, the main advantage of tPCA is that it presents each LEP component with a ‘clean’ shape, extracting and quantifying it free of the influence of adjacent or subjacent components. This property is of particular interest in studies of emotional/attentional processing, where the use of PCA techniques was previously recommended because of their ability to disentangle and characterise LEP components (e.g. [52]). In brief, the tPCA computes the covariance between all LEP time points, which tends to be high between those time points involved in the same component and low between those belonging to different components. The solution is therefore a set of different factors made up of highly covarying time points, which ideally correspond to LEP components. The tPCA based on a covariance matrix was performed on the averaged waveforms, each being represented by 300 time-digitised points (from 200 to 1000ms averaged epoch). Forty-two subjects, six trial categories (3 emotional pictures and 2 laser stimulus) and sixty electrode sites yielded a total of 15,120 averaged waveforms which served as the data base for the PCA. The decision on the number of factors to extract was made by applying a screen test [53]. Selected factors were Promax rotated, as recently recommended [54,55].

**Fig 2:**
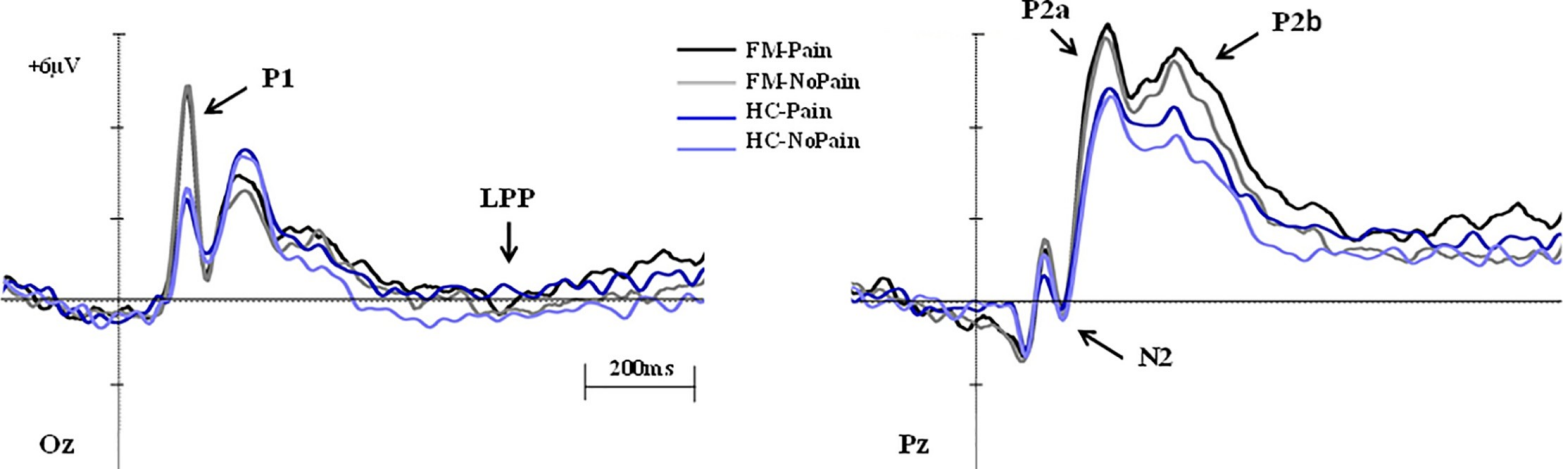
Grand averages corresponding to fibromyalgia (FM) and healthy control (HC) participants in response to Pain and No pain conditions. Scales and polarity are shown at central and posterior areas.

Statistical analyses were performed on SPSS Statistics 22.0 (IBM, Inc). Based on both the PCA analysis and grand averages inspection (see Fig 2), pertinent LEP time windows were selected for analysing different phases within the pain processing. The mean amplitude was calculated for each LEP component, choosing nearby electrodes regions, in each temporal window. A 3 × 2 × 2 repeated-measures ANOVAs were carried out including Emotion (N, A-, P), Laser stimulus (Pain, NoPain) and Group (FM patients and HC participants) as the between-subject factor in each LEP time window. The Greenhouse–Geisser (GG) epsilon correction was applied to adjust the degrees of freedom of the F ratios where necessary, and post hoc comparisons to determine the significance of pairwise contrasts were performed using the Bonferroni procedure (alpha < .05). Effect sizes were also reported using the partial η -square (η^2^_p_) method where significant contrasts occurred. Relationships between LEP amplitudes and psychological measures (STAI, BDI and PCS) were also tested by means regression analyses. As with the behavioural analyses, here too we tested the possible effect of the various medications on the FM group through a one-way analysis of variance model.

#### Source-estimation

In order to explore the cortical regions that might account for the experimental effects, standardised, low-resolution brain electro-magnetic tomography (sLORETA: [56]) was applied to relevant LEP amplitudes according to the ANOVA results. sLORETA is a 3D, discrete, linear solution for the EEG inverse problem [57], which refers to a three-shell spherical model registered to the MNI305 digitised, structural human brain atlas template. Solutions are therefore given in three co-ordinates: ‘x’ is the distance in millimetres to the right (+) or left (−) of the midline, ‘y’ is the distance anterior (+) or posterior (−) to the anterior commissure and ‘z’ is the distance above (+) or below (−) a horizontal plane through the anterior and posterior commissures. Although it is generally recommended that EEG-based source estimation solutions be interpreted with caution, due to their potential error margins, LORETA solutions have shown significant correspondence with those provided by haemodynamic procedures in the same tasks [58,59].

In its current version, sLORETA computes the current density at each of 6,239 voxels mainly located in the cortical grey matter of the digitised, Montreal Neurological Institute (MNI) standard brain. Therefore, to identify brain regions underlying different phases of pain processing, a two-step analysis was carried out for the LEP components that were sensitive to experimental manipulation. First, three-dimensional current–density estimates for relevant LEP amplitudes were computed for each participant and each experimental condition. The voxel-based, whole-brain sLORETA images were compared among the six experimental conditions (N Pain, N NoPain, A- Pain, A- NoPain, P Pain and P NoPain) using the non-parametric mapping (SnPM) tool in the sLORETA software package. This non-parametric methodology inherently avoids multiple comparison-derived problems and does not require any assumption of normality (for an explanation of it, see [60]). The next step was based on a region-of-interest (ROI) approach. Thus, voxels that showed significant differences between experimental conditions (log-F-ratio statistic, two-tailed corrected p < 0.05) were located in specific Brodmann areas (BAs). Subsequently, current densities of these ROIs (radius = 5 mm) were subjected to ANOVAs using Emotion (three levels: N, A− and P), Laser stimulus (Pain and NoPain) and Group (FM and HC) as factors.

http://dx.doi.org/10.17504/protocols.io.zhtf36n [PROTOCOL DOI]

## Results

### Control results and picture detection test

Intensities (mJ/mm^2^) of laser stimuli used for each group of participants were analysed by t- test. Differences between threshold levels were found for both supra-threshold level (FM group: M = 21.56, SD = 3.56; HC group M = 21.30, SD = 2.00) and infra-threshold level (FM group M = 31.38, SD = 7.59, HC group M = 31.74, SD = 5.10). Specifically, higher intensities had to be used for supra-threshold stimulus compare infra-threshold level (t = 0.286, p = .013). No differences were found for group of participants.

Participants’ assessments of the valence and arousal of emotional pictures were computed using repeated-measures ANOVAs. Analyses yielded significant differences in both Valence [F(2,39) = 453.341, p < .000, η^2^_p_ = .919] and Arousal [F(2,39) = 195.479, p < .000, η^2^_p_ = .830]. Post hoc contrasts indicated that the three emotional pictures differed from each other on both scales. Whereas A- pictures were evaluated as the most negative and arousing, N pictures were assessed as more neutral and less arousing. Furthermore, FM patients classified P pictures as more negative than did those in the HC group [F(1,40) = 8.544, p = .006, η^2^_p_ = .176]. No differences were found for other comparisons. Mean values for Valence and Arousal relating to the three emotional categories (separated by group of participants) are displayed in Table 2.

With respect to the picture detection test, subjects were indeed subjectively unaware of any element included in the masked pictures (only in 31% of trials did subjects say *yes* to the first question). The d′ parameter of SDT was calculated for each participant after calculating the hit and false alarm rates. The mean for the whole sample was d′ = -0.64. As mentioned before, d’ values lower than 1 indicate non-awareness discrimination for masked pictures. All these results support the validity of the selected pictures and masking method for use in subsequent analyses.

### Behavioural results

Mean values for PR and RTs relating to the influence of emotional masking stimuli on each type of Laser stimulus (separated out by group of participants) can be seen in Table 3. We carried out three-way repeated-measures ANOVAs 3 × 2 × 2 on these two variables (PR and RTs), including group as the between-subject factor. A significant main effect related to the type of Laser stimulus was found [F (1,40) = 143.905 p < .001, η^2^_p_ = .782]. As expected, painful stimuli (M = 1.74, SD = 0.489) elicited higher PR than did non-painful stimulation (M = 1.36, SD = 0.391). However, our analyses revealed no other effect with reference to group or emotional pictures (F<1). In the case of RT, both main and interaction effects between the aforementioned factors were also tested, but none reached statistical significance (F <1).

**Table 3:** Mean values for PR and RTs.

Behaviour	N Pain	N NoPain	A- Pain	A- NoPain	P Pain	P NoPain
PR FM	1.71 (0.30)	1.32 (0.33)	1.70 (0.35)	1.34 (0.32)	1.72 (0.41)	1.31 (0.25)
PR HC	1.78 (0.58)	1.38 (0.43)	1.81 (0.60)	1.41 (0.49)	1.76 (0.57)	1.41 (0.44)
RT FM	1182.88 (212.30)	1178.67 (240.69)	1192.92 (242.52)	1187.21 (239.87)	1191.35 (215.86)	1042.77 (206.46)
RT HC	1061.62 (214.22)	1075.20 (244.78)	1083.90 (203.72)	1076.47 (230.02)	1188.70 (236.56)	1080.53 (232.76

Means and standard deviations (in parenthesis) relating to pain rating (PR) and reaction time (RT) for each emotional picture and pain condition by group.

Finally, stepwise regression analyses were carried out for the FM group in the painful trials. However, no significant predictors for behavioural indices (PR and RT) were found with respect to trait anxiety (β = -0.028, p > .05; β = -0.016, p > .05), depression (BDI scores) (β = -0.053, p > .05; β = -0.055, p > .05) or the Pain Catastrophizing Scale (β = -0.055, p > .05; β = -0.026, p > .05). Only in the case of state anxiety (β = -0.030, p > .05; β = 0.162, p = .044), was a linear association presenting a positive slope found for RT: the higher the state anxiety, the longer the RT. Specifically, this effect was only present for Pain-related (β = 0.176, p = .037) and Negative pictures (β = 0.201, p = .027). ANOVAs checking for the effect of medication-taking on PR and RT in FM patients showed no statistical differences (analgesics F(1,18) = 2.238, p > .05; F(1,18) = 0.048, p > .05, NSAIDS F(1,18) = 2.062, p > .05; F(1,18) = 0.210, p > .05, tricyclics F(1,18) = 0.000, p > .05; F(1,18) = 0.348, p > .05, SSRI F(1,18) = 2.614, p > .05; F(1,18) = 0.019, p > .05 and benzodiazepines F(1,18) = 0.147, p > .05; F(1,18) = 0.963, p > .05).

### LEP results

Fig 2 shows a selection of grand averages once the baseline value (pre-stimulus recording) had been subtracted from each LEP. These grand averages, characterising P1, N2, P2a and P2b, correspond to the scalp sites where interaction effects between Laser stimulus and Group of participants were most clearly observed, as explained later.

#### Detection and characterisation of LEP components

**Fig 3:**
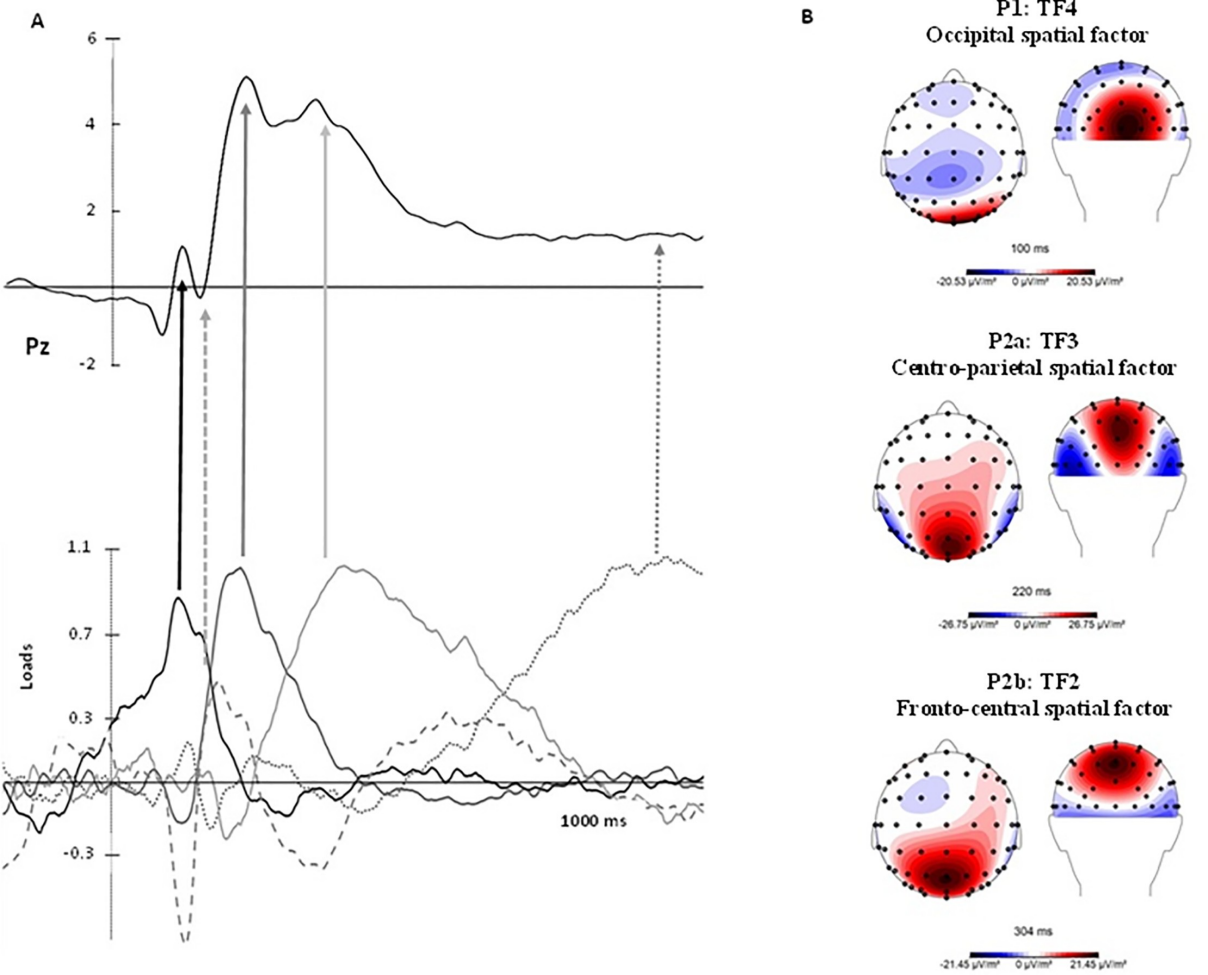
Correspondence between LEP components and TFs derived from the tPCA. This figure shows: A) tPCA and the correspondence of each component in the grand average in Pz; and B) maps showing the topographical distribution of the P1 (TF4), P2a (TF3) and P2b (TF2) components, where experimental effects were found. Red areas reflect greater activity.

As a consequence of the tPCA, five temporal factors (TFs) were extracted from the LEPs and submitted to promax rotation (see Fig 3 for the correspondence between LEP components and TFs derived from the tPCA). Extracted factors explained 73.65% of the total variance (50.27%, 9.75%, 6.31%, 4.33% and 2.97%, respectively). Regarding their peak latency and topography distribution of TF2, TF3 and TF4 (peaking at 380, 200 and 100ms at centro-parietal, fronto-central and occipito-parietal sites of the scalp) were identified and associated with the components signalled in the grand averages as P2b, P2a and P1, respectively (Fig 3). In the case of P2, some authors report that this component usually shows a second peak and focus their statistical analyses on the greater one only [61]. However, on the basis of our tPCA results (P2 was separated in two factors: TF2 and TF3) we decided to analyse both of them, calling the first peak P2a and the second one P2b. Furthermore, TF1 (peaking around 820ms; central sites) and TF5 (peaking at 154ms; parieto-occipital sites) were related to late positivity potential (LPP) and N2, respectively.

#### LEP analyses: Experimental effects

Given our objectives, we tested the influence of subliminal emotional pictures on pain processing in FM patients. In accordance with peak latency data corresponding to the TFs extracted from the tPCA, 3 × 2 × 2 repeated-measures ANOVAs focused on the amplitudes of LEP components elicited by trials containing Emotional pictures and Laser stimulus (A- Pain, A- NoPain, N Pain, N NoPain, P Pain and P NoPain) with respect to Group (two levels: FM and HC participants), as previously described. Thus, pertinent LEP time windows were selected, choosing nearby electrodes regions (see Table 4). Mean amplitude was obtained at the 80–120ms window for P1, the 130–170ms window for N2, the 190–270ms window for P2a, the 280–360ms window for P2b and the 500–920ms window for LPP. Statistical details of these analyses on the LEP components reaching statistical significance are shown in Table 4.

**Table 4:** Description and statistical results for P1, P2a and P2b LEP components.

Temporal Factor	Peak (ms)	Scalp distribution	ANOVAs Emotion d.f. = 2, 39	ANOVAs Laser stimulus df = 1, 40	ANOVAs Group d.f. = 2, 39	ANOVAs Interaction Emotion x Laser stimulus x Group, df = 2, 39
TF4 (P1)	100	O1, Oz, O2, POz, PO3, PO4	F = 1.627, p = .203	F = 0.950, p = .336	**F = 4.789, p = .035**	**F = 3.387, p = .039**
TF3 (P2a)	200	C1, Cz, C2, CP1, CPz, CP2, CP4	F = 1.048, p = .356	F = 0.019, p = .890	**F = 9.949, p = .003**	F = 4.358, p = .066
TF2 (P2b)	380	FC1, FCz, FC2, C1, Cz, C2	F = 0.033, p = .968	**F = 5.853, p = .020**	**F = 7.214, p = .010**	F = 1.355, p = .264

TF, temporal factor; df = degrees of freedom.

As can be observed in Table 4, a significant Group effect was found for P1, showing higher amplitudes for the FM than for the HC group. Interestingly, an interaction effect including Emotional picture by Laser stimulus by Group was also found for this LEP component. Post hoc comparisons indicated that P1 showed greater amplitudes in response to painful stimulus when it was preceded by pain-related pictures, compared with the rest of the emotional conditions. This effect was only found for the FM group. For P2a and P2b components, the same main effect of Group was found as for P1. In the case of P2b, (an LEP component traditionally associated with pain processing), a main effect of Laser stimulus was found, where the painful condition generated higher amplitudes than those observed in the painless condition. No other significant effects were found on N2 or LPP components. Finally, FM patients who were taking medication showed no differences in P1 amplitudes (analgesics F(1,18) = 2.000, p > .05, NSAIDS F(1,18) = 0.149, p > .05, tricyclics F(1,18) = 1.203, p > .05, SSRI F(1,18) = 1.166, p > .05 and benzodiazepines F(1,18) = 1.482, p > .05); P2a (analgesics F(1,18) = 0.270, p > .05, NSAIDS F(1,18) = 0.013, p > .05, tricyclics F(1,18) = 0.185, p > .05, SSRI F(1,18) = 3.937, p > .05 and benzodiazepines F(1,18) = 1.173, p > .05); or P2b (analgesics F(1,18) = 0.093, p > .05, NSAIDS F(1,18) = 0.012, p > .05, tricyclics F(1,18) = 1.245, p > .05, SSRI F(1,18) = 0.010, p > .05 and benzodiazepines F(1,18) = 0.330, p > .05), compared with FM patients not undergoing clinical treatment.

Stepwise regression analyses were also carried out in the FM group. Again, no significant predictors for P1, P2a and P2b amplitudes were found for trait anxiety (β = 0.032, p > .05; β = 0.049, p > .05), state anxiety (β = 0.029, p > .05; β = 0.010, p > .05; β = 0.145, p > .05), scores on the BDI (β = 0.016, p > .05; β = 0.020, p > .05; β = 0.002, p > .05) or the Pain Catastrophizing Scale (β = 0.010, p > .05; β = 0.014, p > .05; β = 0.047, p > .05). A linear association showing a positive slope was found for P2a (β = 0.261, p = .021) for higher trait anxiety higher amplitudes―specifically, when a negative picture preceded the pain (β = 0.369, p = .004). A positive linear association with state anxiety was also found for P2b LEP, but this effect was only present for the P Pain condition (β = 0.251, p = .024).

#### Source estimation

**Fig 4:**
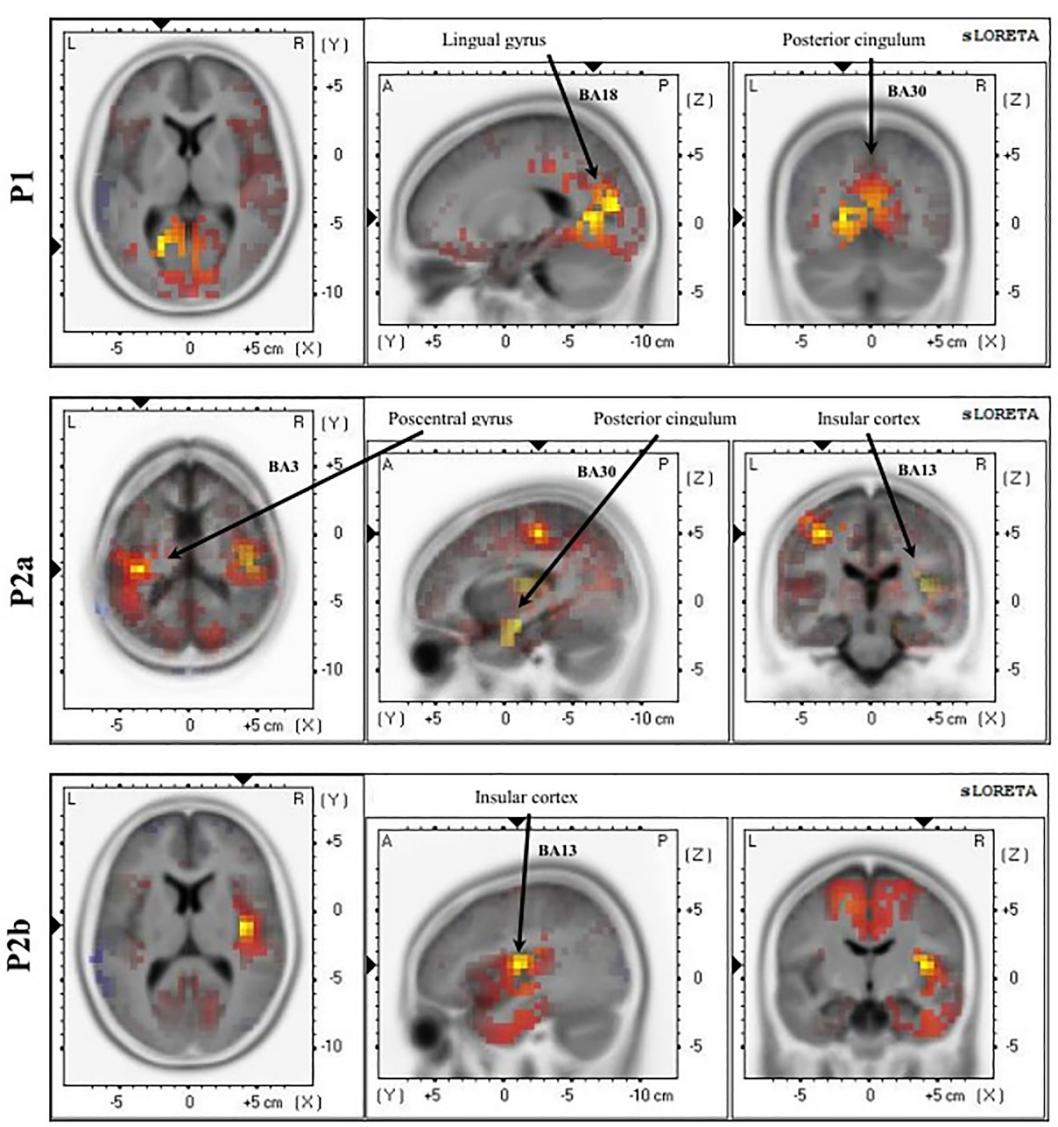
sLoreta solution for main ROIs relating to scalp LEP components: P1, P2a and P2b.

In order to identify the neural sources relating to the experimental effects observed at the scalp level, sLORETA maps were computed for each participant and experimental condition in relevant LEP amplitudes of P1, P2a and P2b. Subsequently, and as we explained in the Methods section, several ROIs were defined according to data-driven criteria (see Fig 4). Current densities associated with each ROI were quantified and subjected to ANOVAs. In Table 5, the full statistical effects for ROIs activity relating to each LEP component with respect to group and laser manipulations, can be observed.

**Table 5:** Description and statistical results for the ROIs obtained for P1, P2a and P2b.

LEP	ROI	ANOVAs (Laser stimulus, df = 1, 40)	ANOVAs (Group, df = 2, 39)	Effect
**P1**	Lingual gyrus (BA18) x = -25, y = -98, z = -26	F = 0.831, p = .368	**F = 7.134, p = .011**	FM > HC
	Posterior cingulum (BA30) x = -20, y = -65, z = 5	F = 0.013, p = .908	**F = 12.527, p = .001**	FM > HC
**P2a**	Postcentral gyrus (BA3) x = -35, y = -25, z = 50	**F = 6.050, p = .018**	F = 0.087, p = .769	Pain > NoPain
	Insular cortex (BA13) x = 45, y = -15, z = 15	F = 0.021, p = .886	**F = 14.780, p < .000**	FM > HC
	Posterior cingulum (BA30) x = 15, y = -63, z = 13	F = 0.015, p = .903	**F = 4.156, p = .048**	FM > HC
**P2b**	Insular cortex (BA13) x = 40, y = -10, z = 10	**F = 11.100, p = .002**	**F = 5.576, p = .023**	FM > HC Pain > NoPain

This table shows the statistical results of the ROIs linked to each LEP. Brain region denomination and Broadmann area (BA), as well as the direction of the statistical effect, are described. df = degrees of freedom.

According to the computed comparisons, three brain regions showed significant differences by group (see Table 5). Post hoc analyses showed greater cortical activity in the FM group than in the HC group within the Lingual gyrus (BA18), posterior cingulum (BA30) and insular cortex (BA13). In addition, two ROIs were sensitive to laser stimulus (postcentral gyrus -BA3- and insular cortex -BA13-), where maximal current densities were used for pain stimulation.

## Discussion

The aim of the present study was to explore neural correlates relating to the influence of visual masking emotional stimulation on pain processing in fibromyalgia patients. Brain activity results showed that the emotional charge conveyed by subliminal pictures modulated pain responses in these patients. In particular, masking pain-related pictures enhanced pain processing, as reflected in higher P1 amplitudes. Additionally, activity within occipital and limbic regions (the lingual gyrus and PCC) showed higher activation in the fibromyalgia group compared with the healthy control group. In accordance with the previous literature [44,62], our LEP analysis showed the presence of a classical N2-P2 complex in response to CO_2_ laser stimulation. P2a and P2b presented greater amplitudes for fibromyalgia patients than for healthy controls. Furthermore, P2b amplitude was sensitive to the intensity of laser stimulation, being higher for painful than for painless events. In addition to the LEP data, the activity detected within the insular cortex, postcentral gyrus and posterior cingulum was also strongest for pain conditions and fibromyalgia patients, compared with the other stimuli and the control group, respectively. However, we did not observe any behavioural effect relating to the emotional modulation of pain. A careful interpretation of our results is given as follows.

As we have already indicated in the Results section, an early positive wave peaking around 100ms (P1) after laser stimulus reflected an emotional modulation effect, in which painful stimulation preceded by pain-related pictures elicited higher P1 amplitudes than did painful trials preceded by any other kind of emotional picture. Only patients with fibromyalgia were sensitive to such unconscious emotional modulation. Although it might seem an early response to pain, previous pain studies [62–64] have indicated that LEP might appear at latencies of around 100–150ms, displaying a triphasic morphology (W-shaped waveform) and delineating four major components (N1, P1, N2, P2). The peak latencies and amplitudes of these components can vary, depending on both the measurement parameters (e.g., laser intensity stimulation, duration, etc) and experimental conditions [62]. Recent studies using laser stimulation on the dorsum of the hand have described a P1 component at central scalp regions with a peak latency ranging between 97–204 ms [65] that may even vary as a function of the body part stimulated [34]. Although the central scalp distribution of the P1 detected here is in line with data reported by previous LEP studies [65], the emotional modulation effect on posterior P1 scalp regions has not previously been described.

The P1 component (LEP relating to pain processing) has also been associated with exogenous attentional processes to physical components of somatosensory stimuli and the possible overcoming of a painful stimulus [66,67]. Some studies have suggested that this early component reflects pain cortical processing preceding the conscious evaluation of stimuli intensity. Both requirements are linked to the visual masking paradigm, where pain stimulation preceded by masking emotional pictures and prominent P1 amplitudes for fibromyalgia patients in trials involving pain-related pictures would lead us to think that relevant subliminal information has an effect on exogenous attention contributing to a more intense processing of painful stimulation in these patients. Similar modulations have been previously reported using visual masking paradigms where masked emotional faces led to an enhancement of early ERPs amplitudes compared to neutral faces [68]. Therefore, that early modulation might reflect processes involved in a rapid detection of emotionally significant sensory stimuli, even when such signals are insufficient to result in perceptual awareness. Regarding current data, this rapid and efficient detection of potentially threatening signals would lead to an augmented processing of painful stimulus in patients with fibromyalgia. Clinical studies have also shown that chronic pain patients selectively attend to both pain sensory and pain affective stimuli [69], suggesting that early responses to them could be the result of automatic processes (i.e., biases towards pain-related stimuli) rather than conscious control [30]. Similarly, priming studies have indicated that affective pain-related primes increase the processing of painful stimuli in chronic pain patients, as reflected by higher amplitudes in LEPs [61]. Interestingly, subliminal paradigms have confirmed the ability of emotional pictures primes to modulate LEPs, suggesting that such brain waves could be a neural index of pain-memory network activation in patients, despite not observing an increase in the perception of pain [21]. Our results reinforce the idea that pain-related information, even when it is unconsciously perceived, can enhance attentional resources, increasing the neural activity involved in processing painful stimulation. Although previous studies suggest that an enhancement of exogenous attention to pain-related information might be the first step to activate pain memories [20–22], the lack of an augmented pain perception in fibromyalgia patients lead us interpreted present data with caution.

Additionally, at the neural level, two sources were linked to P1 scalp activity. ROIs’ current densities in both the lingual gyrus and PCC (BAs 18/30) showed an enhancement for fibromyalgia compared with control participants. Whereas brain-imaging studies have recently implicated the lingual gyrus in emotional processing and visual recognition, the role played by the posterior cingulate cortex (PCC) is associated with certain aspects of pain processing [70,71]. Activity within the PCC has been linked to visuospatial functions as a part of defence responses aimed at avoiding potentially unpleasant stimuli [72]. In this vein, Vogt and coworkers [73] recorded early responses within the PCC (including the caudal cingulate motor area) to somatosensory events, suggesting that this region might be related to reflexive reactions (as both a defence response and orientation response) to unpleasant stimuli. Given that the region analysed here has reciprocal and profuse connections with others closely involved in both affective and pain processing―such as the orbitofrontal and anterior cingulate cortices―it has been suggested that the PCC might be associated with processes of emotional assessment of events that are relevant to an individual [73–75]. An increase in activation in this region has been observed in chronic pain patients when faced with negative visual stimulation compared with neutral and positive emotional stimuli, leading to the suggestion that this enhanced activity in the PCC might be linked to higher processing of aversive aspects of pain [76]. Recent studies have proposed that the functional role of the PCC might be more closely related to the processing of psychological pain components rather than to the physical or sensorial processing of the painful stimulus. [76,77].

Various sources of evidence suggest that increased processes of exogenous attention depend on the kind of stimulus employed during the experimental task: words, pictures or faces [29,78,79]. In this vein, it has been suggested that words are not sufficiently able to activate pain memories because they do not depict the threatening content conveyed by the stimulation as effectively as emotional pictures can [28]. In contrast, pictorial non-word stimuli have been reported as being more arousing and intrusive than words [28,80] and consequently more capable of capturing attention [24,25], eliciting attentional biases and modulating the processing of further stimulation. Our results indicate that only pictorial information closely related to the main concerns of fibromyalgia patients (i.e. pain-related stimuli) was capable of intensifying pain processing, eliciting early brain responses even when individuals were unaware of this stimulation. However, the lack of behavioural effects on pain perception in fibromyalgia do not allow to confirm its capability to activate pain memories, so this issue is still under debate.

In the current study, LEPs in response to laser stimuli applied just after the presentation of subliminal emotional pictures showed the typical waveforms described in other studies using conscious stimulus [16,61]. These comprised a negative peak at around 150–260ms (N2), and a subsequent positive peak at around 250–400ms (P2). In line with our results, the amplitude of the P2b component (or the second peak of P2) shows good correlation with pain intensity, this being higher for painful conditions than painless ones. Although this component showed classic pain sensitivity (higher for painful than for painless stimuli), the modulation effect of emotion on pain processing was not observed in P2, in contrast with other experiments showing higher amplitudes for painful stimuli after negative or pain-related primes [16,33,81]. This effect has been found consistently in supraliminal emotional studies, but not when subliminal presentation was used [21,22,25]. Nonetheless, it should be remarked that these kinds of studies usually only focus on the most studied laser-evoked components (N2-P2). In the present study the whole LEP response was explored, revealing the above-mentioned early emotional modulation in P1. Hence, it has been proposed that subliminal processes have a pre-attentive origin, triggering responses that are important in generating an immediate response to relevant and potentially threatening stimuli [82]. Thus, it might be suggested that relevant subliminal stimuli can modulate early latency brain responses linked to exogenous attention and automatic processes, but not late latency waves, which are more closely related to endogenous attention and controlled processes. Additional studies on this subject should be conducted using an LEP technique with high sensitivity. Interestingly, enhanced amplitudes in the P2a and P2b LEP components were observed in fibromyalgia subjects compared with control participants, as in previous studies [31,34], suggesting that patients devote greater cognitive processing to painful stimulation. Furthermore, the fact that greater P2a/P2b amplitudes were related to high levels of trait and stated anxiety in FM group suggest the presence of a hypervigilance response in FM patients, as described in many studies [83,84].

With regard to the analyses carried out at neural level, two sources associated with P2 scalp activity (i.e., the insular cortex and PCC) recruited the greatest neural activity for fibromyalgia patients compared with the control group. The insular cortex has been widely described as part of the pain processing cerebral system or ´pain matrix´ [4,85]. The role played by these cortical regions in pain processing is further supported by neuroimaging studies, in which an augmented activation pattern has been demonstrated in patients with fibromyalgia [86]. Furthermore, greater connectivity between the insular cortex and orbital cortex and the attentional network is apparent in these patients [87]. The insular cortex has been identified as coding both intensity and pain localisation; however, it is also involved in connection with the cingulate cortex in automatic reactions, affective-motivational functions and the association of emotions with former painful experiences, and it contributes to the negative expectancy effects of pain [88,89]. It has been observed that this augmented activation is not only apparent for painful stimulation, but also for non-painful events [86], as our data also show. These results support the hypothesis of Brown and co-workers [89], which postulates that an abnormal insular cortex response is a common mechanism for pain processing in chronic pain patients.

Although consistent differences were found in cerebral activity between the fibromyalgia and healthy control groups, our behavioural results showed no group differences associated with the application of laser stimulation (i.e., pain intensity rating and reaction times). Although augmented pain perception might be expected in trials involving pain-related primes, previous studies have shown mixed results in this regard. Thus, whereas some authors have found lower pain tolerance related to threatening priming conditions [22], a lack of difference has been reported in other studies using emotional subliminal stimulation to modulate pain experience [21,24,25]. Nonetheless, LEPs amplitudes and pain perception not always have a straightforward relationship. LEP components (P1, P2a/P2b) are generated by a network of brain areas, so correlation between both indices probably only may understood by conducting more complex analysis. Because of that, it should be noted that pain ratings measures are the final single output of a large set of neural processes that may not always be convergent, being highly dependent on experimental conditions. For that reason, an advantage of using LEPs is that components can be examined in the absence of an overt behavioural response, and our results are a good example of the greater sensitivity of LEPs to the effects of subliminal emotional stimulation in pain processing.

The current study does, however, have some potential limitations that need to be addressed. Given that the negative picture condition was made up of stimuli belonging to various negative categories of emotion, such as sadness, fear and disgust, future studies should take this into account. Recent studies [90,91] have indicated that the brain’s responses to negative emotional stimuli (i.e. fear and disgust) might differ even when they share similar levels of valence and arousal. In fact, disgusting events appear to be more efficient at attracting exogenous attention than do other negative emotions. Another issue to take into account relates to the pharmacological treatments taken by patients. Although the influence of certain medications (i.e., ketamine, benzodiazepines) on higher-order cognitive functions has been well described [92,93], its impact on more automatic process such as attention or implicit memory [94,95] is less well understood [93,96]. Furthermore, studies comparing medicated and non-medicated patients found no differences in the results, probably because the low doses being taken were not sufficient to cause any untoward effect [97]. In light of the results obtained in our study, pain perception and exogenous attention do not seem to be affected by psychoactive drugs. Nevertheless, tighter control over this variable might be an improvement for future studies. Finally, another aspect to consider is that only female participants were included in the current study. Although the disease is more prevalent in women than in men [98], some recent studies have indicated that the female/male proportion is not so different, being less than 60% in females [99]. In future studies this should also be considered.

In summary, our results show that pain-related pictures are capable of modulating early cerebral responses to pain in fibromyalgia, even when patients are unaware of the emotional content conveyed by such stimuli. This subliminal emotional modulation at early stages of pain processing, as shown by higher P1 amplitudes (at scalp level), indicates greater engagement of automatic attention biased towards pain-related stimuli in fibromyalgia patients, probably leading to an enhancement of pain processing. Enhancement of attention to pain-related primes may be hypothesised as the first step in activating a pain-memory network (chronic pain patients show a lower threshold for the activation of pain concepts in memory). However, this subliminal modulation is not presented on later pain processing stages (i.e., P2 component at scalp level and insular cortex/PCC activity at source neural level), which are more closely related to endogenous attention and controlled processes. Our LEP data represent the first study providing objective evidence that emotional subliminal information can modulate early latency brain responses to pain (i.e. the P1 wave) linked to exogenous attention in fibromyalgia. Further research should be done using LEP methodologies (because of its greater sensitivity) to fully understand unconscious emotional influences on pain processing in patients with fibromyalgia.
